# Trajectory of cardiometabolic disease‐related hospitalizations before and after dementia diagnosis

**DOI:** 10.1002/alz70860_102085

**Published:** 2025-12-23

**Authors:** Sakura Sakakibara

**Affiliations:** ^1^ Aging Research Center, Karolinska Institutet, Stockholm, Stockholm, Sweden

## Abstract

**Background:**

Dementia is associated with increased healthcare use, though the timing of hospitalizations in relation to the development of dementia remains unclear. Cardiometabolic diseases (CMDs) including type 2 diabetes, heart disease, and stroke often co‐occur with dementia, but the impact of dementia on CMD‐related hospitalizations remains unknown. We aimed to map the trajectory of CMD‐related hospitalization before and after dementia diagnosis and to further identify factors of CMD‐related hospitalization.

**Method:**

Within the Swedish Twin Registry, 1657 participants aged ≥65 (58.8% women) with incident dementia were matched with 1657 dementia‐free participants using propensity scores. Participants were followed for up to 20 years to detect CMD‐related hospitalizations. Dementia and CMD‐related hospitalizations (planned and unplanned) were identified from medical records. Factors of CMD‐related hospitalizations included age, sex, education, marital status, smoking, drinking, physical activity, body mass index, and hypertension. Data were analyzed using Poisson regression and generalized estimating equation models.

**Result:**

Compared to the controls, the incidence of CMD‐related hospitalization among people with dementia started to increase from 4 years before (incidence rate ratio [IRR] 1.25, 95% confidence interval [CI] 1.00‐1.57) and started to decrease 3 years after, the diagnosis (IRR 0.74, CI 0.58‐0.94). People with dementia had a greater number of CMD‐related hospitalizations (IRR 1.16, CI 1.04‐1.30), longer hospital stays (IRR 1.19, CI 1.04‐1.56), and higher 30‐day readmissions (IRR 1.27, CI 1.04‐1.56) in the five years before dementia diagnosis, but these rates decreased after the diagnosis. Male sex, smoking, obesity, and hypertension were associated with further increases in CMD‐related hospitalization among people with dementia.

**Conclusion:**

CMD‐related hospitalization changes over the course of dementia development. Our findings suggest that the healthcare burden increases even before the diagnosis of dementia, and CMD‐related hospitalization may be an early sign of the future onset of dementia.

